# 3*β*-Hydroxysteroid-*Δ*24 Reductase (DHCR24) Protects Pancreatic *β* Cells from Endoplasmic Reticulum Stress-Induced Apoptosis by Scavenging Excessive Intracellular Reactive Oxygen Species

**DOI:** 10.1155/2020/3426902

**Published:** 2020-07-16

**Authors:** Yang Li, Xude Wang, Baoyu Yang, Haozhen Wang, Zhenzhong Ma, Ziyin Lu, Xiuli Lu, Bing Gao

**Affiliations:** ^1^Department of Biochemistry and Molecular Biology, Life Science School, Liaoning University, Shenyang 110036, China; ^2^Department of Cell Biology and Genetics, Shenyang Medical College, Shenyang 110034, China

## Abstract

There is accumulating evidence showing that apoptosis induced by endoplasmic reticulum (ER) stress plays a key role in pancreatic *β* cell dysfunction and insulin resistance. 3*β*-Hydroxysteroid-*Δ*24 Reductase (DHCR24) is a multifunctional enzyme located in the endoplasmic reticulum (ER), which has been previously shown to protect neuronal cells from ER stress-induced apoptosis. However, the role of DHCR24 in type 2 diabetes is only incompletely understood so far. In the present study, we induced ER stress by tunicamycin (TM) treatment and showed that infection of MIN6 cells with Ad-DHCR24-myc rendered these cells resistant to caspase-3-mediated apoptosis induced by TM, while cells transfected with siRNAs targeting *DHCR24* were more sensitive to TM. Western blot analysis showed that TM treatment induced upregulation of Bip protein levels in both cells infected with Ad-LacZ (the control group) and Ad-DHCR24-myc, indicating substantial ER stress. Cells infected with Ad-LacZ exhibited a rapid and strong activation of ATF6 and p38, peaking at 3 h after TM exposure. Conversely, cells infected with Ad-DHCR24-myc showed a higher and more sustained activation of ATF6 and Bip than control cells. DHCR24 overexpression also inhibited the generation of intracellular reactive oxygen species (ROS) induced by ER stress and protected cells from apoptosis caused by treatment with both cholesterol and hydrogen peroxide. In summary, these data demonstrate, for the first time, that DHCR24 protects pancreatic *β* cells from apoptosis induced by ER stress.

## 1. Introduction

Type 2 diabetes (T2D) is a metabolic disorder associated with a number of risk factors, including, amongst others, genetic factors, environmental exposure, obesity, and age [1, 2]. It is characterized by hyperglycemia due to the insufficient secretion of insulin, caused by a dysfunction of insulin-secreting pancreatic *β* cells, and decreased insulin sensitivity, caused by insulin resistance [3]. T2D is a chronic and lifelong disease with few clinical treatment options currently available.

In T2D, hyperglycemia often occurs after *β* cells progressively fail to compensate for insulin resistance, and *β* cell failure is a crucial factor in the pathogenesis of T2D [4, 5]. In pancreatic tissues of patients with T2D, reduced cell mass has been observed [6, 7], and there is accumulating evidence that apoptosis is an important mechanism of *β* cell mass loss [6–8]. Therefore, therapeutic methods targeting and attenuating *β* cell apoptosis may be an effective method for the clinical management of T2D.

Insulin is synthesized in the endoplasmic reticulum (ER), the key membranous compartment where newly synthesized secretory and membrane proteins are folded, assembled, and transported. Under normal conditions, the ER maintains a state of equilibrium between protein accumulation and folding capacity. Pancreatic *β* cells have a highly developed ER to meet the high requirements of insulin secretion, and it is critical for *β* cells to maintain their ER homeostasis [5]. However, some pathological processes, such as prolonged insulin resistance [4], gluco/lipotoxicity [9], or the formation of islet amyloid [10], may disturb this homeostasis, leading to a cellular stress response called ER stress. In response to ER stress, an adaptive response called the unfold protein response (UPR) is activated, which is initially beneficial. However, a sustained UPR triggers apoptosis in the absence of effective interventions [5].

ER stress-induced apoptosis has been confirmed to cause *β* cell dysfunction and insulin resistance [11]. Researchers have found that the ER stress-specific apoptotic signaling CHOP/GADD153 pathway is activated in MIN6 cells exposed to elevated levels of lipids as well as in human pancreas sections of T2D subjects [12]. Furthermore, it has been shown that ER stress contributes to the inhibition of insulin receptor signaling and deficiency in Xbox-binding protein-1 (XBP-1), a transcription factor regulating the UPR response in ER stress, and results in insulin resistance in mice [13]. Accumulating unfolded or misfolded proteins during ER stress result in the generation of excessive reactive oxygen species (ROS), triggering oxidative stress. Pancreatic *β* cells are highly sensitive to ROS, and excessive intracellular ROS lead to *β* cell death [14, 15]. Moreover, increased levels of ROS have been linked to *β* cell dysfunction in T2D [16]. Therefore, the ability to induce resistance to both ER and oxidative stress is a common criterion for T2D drug screening, and, for example, metformin, a well-known T2D drug, acts on *β* cells by alleviating oxidative stress and ER stress [17].

3*β*-Hydroxysteroid-*Δ*24 Reductase (DHCR24) is a multifunctional enzyme located in the ER of many tissues [18]. In addition to its well-known role in cholesterol biosynthesis, DHCR24 acts as a comprehensive antiapoptotic protein, which is closely related to its antioxidant ability [19–22]. In previous studies, we have reported that DHCR24 exerts a ROS-scavenging effect and can attenuate apoptosis of mouse neuronal N2A cells and mouse embryonic fibroblast cells (MEFs) induced by ER stress and oxidative stress [22–24]. Genome-wide association studies of T2D have implied an association of DHCR24 with the lipid metabolism, obesity, and T2D [25, 26]. However, the role of DHCR24 in T2D has not been elucidated so far. To clarify the function of DHCR24 in pancreatic *β* cells, we assessed the consequences of DHCR24 overexpression in mouse pancreatic MIN6 cells exposed to ER stress, exploring the underlying molecular mechanisms of potential protective functions. Here, we demonstrate for the first time that following ER stress, DHCR24 overexpression prevents pancreatic *β* cell apoptosis through scavenging of excessive ROS. These findings provide new potential therapeutic avenues for the treatment of T2D.

## 2. Materials and Methods

### 2.1. Cell Line and Reagents

MIN6 cells (a donation of University of Osaka, Osaka, Japan) were incubated in Dulbecco's modified Eagle's medium (DMEM/high glucose) supplemented with 10% fetal bovine serum at 37°C in a humid atmosphere with 5% CO_2_. 100 U/ml penicillin and 100 *μ*g/ml streptomycin were also added to the medium. Tunicamycin (TM) was purchased from Sigma-Aldrich (St. Louis, MO) and dissolved in DMSO. Tunicamycin is a commonly used ER stress inducer which acts by inhibiting N-linked glycosylation. Cells were exposed to 0.5 *μ*g/ml and 1 *μ*g/ml TM, cholesterol (250x) (Gibco, Grand Island, NY, USA), and 1 mM H_2_O_2_ for 24 h. A phase-contrast microscope (IMT-2, Olympus, Tokyo, Japan) was used for acquisition of cellular images.

### 2.2. Adenovirus Infection

The recombinant adenovirus expressing DHCR24 tagged with a c-myc epitope (Ad-DHCR24-myc) used in this publication was constructed previously [23]. As a negative control, we used an adenovirus expressing *β*-galactosidase (Ad-LacZ). MIN6 cells were infected with Ad-DHCR24-myc and Ad-LacZ at a multiplicity of infection (MOI) of 50 PFU/cell for 48 h, followed by treatment with TM. Immunocytochemical analysis with antibody staining against c-myc was used to determine the infection efficiency.

### 2.3. siRNA Interference

MIN6 cells were transfected with small interfering RNAs targeting *DHCR24* (siDHCR24) and a negative control siRNA (siControl) purchased from Dharmacon (Lafayette, United States) with DharmaFECT 1 (Dharmacon) according to the manufacturer's instructions. The target sequences for siDHCR24 were previously published [27]. The RNA interference efficiency was measured using semiquantitative RT-PCR.

### 2.4. Adherent Cell Number Analysis and Apoptosis Detection

We used Trypan Blue Staining (Beyotime, Shanghai, China) to analyze the adherent cell number after TM exposure, as previously published [22]. Images of cells were acquired using a phase-contrast microscope. In accordance with the manufacturer's instructions, we detected apoptosis by the TUNEL method using the *in situ* Apoptosis Detection Kit (Takara, Otsu, Japan).

### 2.5. Immunocytochemical (IC) Analysis

IC analysis was carried out as previously described [22]. Briefly, cells on coverslips were fixed and blocked, followed by incubation with a mouse anti-myc antibody (Santa Cruz Biotechnology, CA, USA) or rabbit anti-cleaved caspase-3 antibody (Cell Signaling, Beverly, MA) overnight at 4°C. The cells were then incubated with secondary antibodies against mouse or rabbit IgG conjugated with Alexa Fluor 488/568 (Molecular Probes, Eugene, OR) for 1 h under the exclusion of light. Microscopic images were acquired using a digital fluorescence microscope (BIOREVO BZ-9000, Keyence, Osaka, Japan). Densitometric analysis was performed with ImageJ software.

### 2.6. Western Blot Analysis

Total cellular proteins were extracted with the RIPA lysis buffer (Beyotime, Shanghai, China). Fifty micrograms (50 *μ*g) of total protein lysate was separated using a 10% SDS-PAGE and transferred to polyvinylidene fluoride membranes (Millipore, Billerica, MA, USA), which were blocked with 5% skimmed milk for 1 h at room temperature. The membrane was incubated with primary antibodies overnight at 4°C and subsequently incubated with horseradish peroxidase-labeled secondary antibodies against rabbit/mouse IgG (Sigma-Aldrich, St. Louis, Missouri, USA) for 1 h at room temperature. We used the following primary antibodies: rabbit anti-actin (Sigma-Aldrich, St. Louis, MO), mouse anti-Bip/GRP78 (BD Biosciences, Bedford, MA), mouse anti-myc (Santa Cruz, CA, USA), mouse anti-ATF6/cleaved ATF6, and rabbit anti-phospho-p38 MAPK (T180/Y182) (Cell Signaling, Beverly, MA). The target bands were visualized with a hypersensitive ECL chemiluminescence kit (Beyotime, Shanghai, China). ImageJ software was used for densitometric analysis.

### 2.7. Detection of Intracellular ROS

We detected intracellular ROS using 2′,7′-dichlorofluorescin diacetate (DCFHDA; Molecular Probes), which is a ROS-sensitive fluorescent dye, as described previously [22]. Briefly, cells on glass coverslips were fixed after TM exposure, followed by incubation with 20 *μ*M DCFHDA in PBS. We captured the fluorescence of DCFHDA using a digital fluorescence microscope. ImageJ software was used to detect the fluorescence intensity.

### 2.8. Cholesterol Measurement in Cells

MIN6 cells were trypsinized and collected in 1.5 ml Eppendorf tubes. An aliquot of the cells was used for cell counting, and the remaining cells were then subjected to the lipid extraction based on the method described by Bligh and Dyer [28]. The chloroform phase of the Bligh-Dyer extraction was transferred to a new tube and air-dried to remove the chloroform. The samples were subsequently placed in a vacuum for 30 minutes to remove any residue organic solvent. The dried lipids were finally dissolved with the cholesterol assay buffer. 3*β*-Hydroxysterol, which determined the content of total sterol, was detected with an enzymatic cholesterol assay kit (Roche Diagnostics, Mannheim, Germany).

### 2.9. Semiquantitative Real-Time PCR

The mRNA levels of DHCR24 were measured by semiquantitative real-time PCR 24 h after infection with siRNAs. Briefly, total RNA was extracted from cells using the RNeasy Mini Kit (Qiagen, Hilden, Germany) and reverse transcribed into cDNA using the QuantiNova Reverse Transcription Kit (Qiagen, Hilden, Germany) according to the manufacturer's instructions. The subsequent PCR reaction was performed with the following PCR primers, using glyceraldehyde 3-phosphate dehydrogenase (GAPDH) as an endogenous reference gene: DHCR24 sense: 5′-GGCACGGCATAGAACAGGTC-3′, DHCR24 antisense: 5′-CACAGGCATCGAGTCATCGT-3′, GAPDH sense: 5′-CGACCACTTTGTCAAGCTCA-3′. and GAPDH antisense: 5′-GGGTCTTACTCCTTGGAGGC-3′.

### 2.10. Statistical Analysis

Each experiment consisted of at least three independent replicates. Statistical analysis was conducted using ANOVA and Bonferroni's multiple *t*-tests. A *p* value of less than 0.05 was considered statistically significant.

## 3. Results

### 3.1. Upregulation of DHCR24, Driven by Adenovirus Overexpression, Protects against ER Stress-Induced MIN6 Cell Death

**Figure 1:**
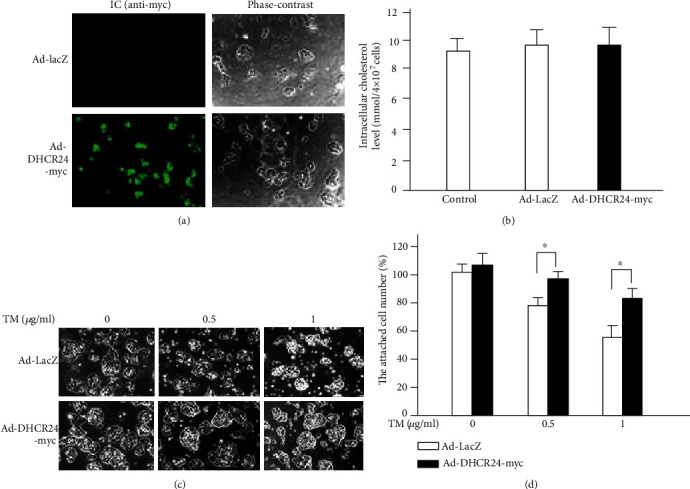
DHCR24 overexpression protects MIN6 cells from cell death caused by TM exposure. (a) Immunocytochemical (IC) analysis against c-myc (green fluorescent signal) was used to determine the transduction efficiency of Ad-DHCR24-myc. Scale bar, 100 *μ*m. (b) Sterol contents in MIN6 cells were measured 48 h after infection with the indicated adenoviruses (50 PFU/cell). Mean ± S.D. (*n* = 3). (c) Images of MIN6 cells were acquired with a phase-contrast microscope 24 h after TM exposure (0, 0.5, and 1 *μ*g/ml). Scale bar, 100 *μ*m. (d) Numbers of adherent cells are expressed as percentage of cells in the Ad-LacZ group at 0 *μ*g/ml TM exposure. Mean ± S.D. (*n* = 3); ^∗^*p* < 0.05 vs. Ad-LacZ.

To investigate the effect of DHCR24 on MIN6 cells, we infected MIN6 cells with Ad-DHCR24-myc or Ad-LacZ (as a negative control). The efficiency of adenovirus infection was confirmed by IC analysis using anti-myc antibody staining. We observed nearly a 100% infection efficiency of Ad-DHCR24-myc infection in MIN6 cells and confirmed that DHCR24-myc was mainly present in the cytoplasm of infected cells (Figure 1(a)).

As DHCR24 catalyzes desmosterol to cholesterol during the last step of cholesterol biosynthesis, we next investigated whether the intracellular cholesterol levels were affected by infection with Ad-LacZ or Ad-DHCR24-myc. There was no significant difference in the cholesterol levels of Ad-DHCR24-myc cells compared to control cells (Figure 1(b)), suggesting that overexpression of DHCR24 did not alter the intracellular cholesterol levels. This may be explained by the fact that intracellular cholesterol concentration is tightly regulated to achieve its hemostasis, because there is an extracellular cholesterol supply from the serum of the culture medium of cells.

In the present study, TM was used to induce ER stress. We observed that the majority of the MIN6 cells in the Ad-DHCR24-myc group survived for at least 24 h after 1 *μ*g/ml TM exposure, while the number of cells in the Ad-LacZ group decreased in a dose-dependent manner with increasing TM concentration (Figures 1(c) and 1(d)). Moreover, the number of lost cells in this group was significantly higher than that in the Ad-DHCR24-myc group, implying that an upregulation of DHCR24 may increase resistance to ER stress-induced death of pancreatic *β* cells.

### 3.2. Overexpression of DHCR24 Inhibits Caspase-3-Mediated Apoptosis

**Figure 2:**
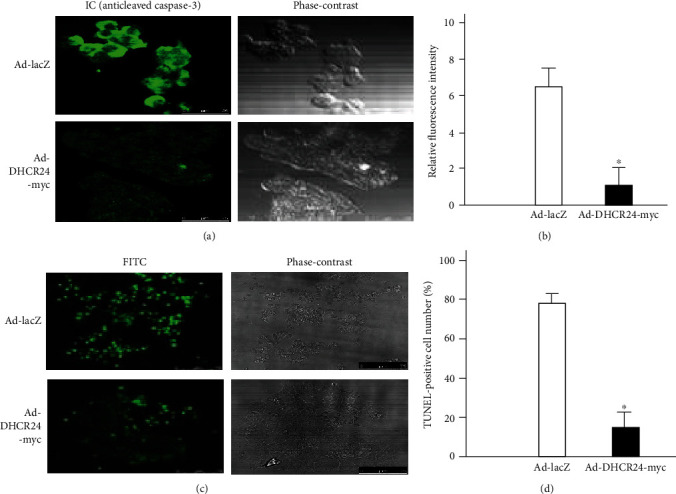
Apoptosis of MIN6 cells induced by TM is inhibited by overexpression of DHCR24. (a) A representative fluorescent image of cleaved caspase-3 (green) was obtained by IC analysis 24 h after TM exposure. Scale bar, 50 *μ*m. (b) The relative fluorescence intensity of cleaved caspase-3 was analyzed with ImageJ software. Mean ± S.D. (*n* = 3); ^∗^*p* < 0.05 vs. Ad-LacZ. (c) The apoptosis of MIN6 cells was analyzed by TUNEL 24 h after TM treatment. The green fluorescent signal represents TUNEL-positive cells. Scale bar, 100 *μ*m. (d) The TUNEL-positive cell number was calculated as percentage of TUNEL-positive cell numbers to adherent cells in the same field. Mean ± S.D. (*n* = 3); ^∗^*p* < 0.05 vs. Ad-LacZ.

Caspase-3 is synthesized as a proenzyme without activity and is cleaved into active fragments in response to apoptotic triggers through self-proteolysis and/or cleavage by other upstream proteases such as caspase-8, caspase-9, and caspase-10 [8, 29, 30]. Therefore, analysis of activated (cleaved) caspase-3 is a common method to identify apoptosis. To assess whether TM-induced cell death of MIN6 was caused by apoptosis, adenovirus-infected cells seeded on coverslips were analyzed by IC by anti-cleaved caspase-3 antibody staining. As shown in Figures 2(a) and 2(b), cells with intense green fluorescent signals in the Ad-LacZ group were significantly more than those in the Ad-DHCR24-myc group, suggesting that the activation of caspase-3 was suppressed by overexpression of DHCR24. This result implied that overexpression of DHCR24 could block the initiation and progression of TM-induced apoptosis in pancreatic *β* cells. Similarly, TUNEL analysis demonstrated that 24 h after TM (1 *μ*g/ml) treatment, about 80% of Ad-LacZ-infected cells were apoptotic, but Ad-DHCR24-myc-infected cells were more resistant to TM-induced apoptosis (Figures 2(c) and 2(d)). Taken together, these data suggested that DHCR24 could inhibit caspase-3-mediated apoptosis in pancreatic *β* cells under ER stress conditions.

### 3.3. Upregulation of DHCR24 Potentiates ATF6 Activation and Inhibits p38 Phosphorylation during UPR in MIN6 Cells

**Figure 3:**
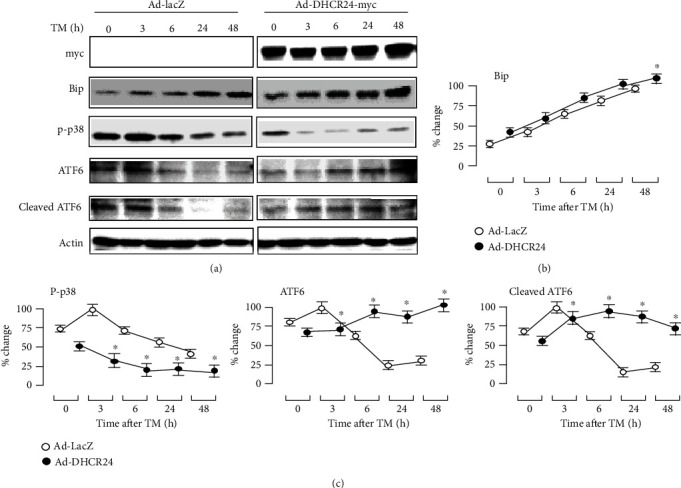
UPR-related signaling pathways were detected after TM treatment by Western blot analysis. (a) Representative Western blots show the expression of c-myc, Bip, p-p38, ATF6, and cleaved ATF6 in MIN6 cells after TM treatment. The levels of these proteins were normalized to actin levels. (b, c) The levels of Bip, p-p38, ATF6, and cleaved ATF6 were calculated as percentage of the largest level in the negative control (Ad-LacZ) group. Mean ± S.D. (*n* = 3); ^∗^*p* < 0.05 vs. Ad-LacZ.

The overexpression of DHCR24 induced by Ad-DHCR24-myc in MIN6 cells was also demonstrated by Western blotting analysis as shown in Figure 3(a). Bip (immunoglobulin heavy-chain binding protein, also known as Grp78) is an ER chaperone that helps proteins fold, assemble, and transport correctly and is upregulated and activated during ER stress [12]. Therefore, Bip can be used as an indicator of ER stress. Using Western blotting, we found that lso demonstrated the overexpression of DHCR24 induced by Ad-DHCR24-myc and Bip levels increased progressively in MIN6 cells after TM treatment, indicating that TM induced ER stress (Figures 3(a) and 3(b)). Cells in the Ad-DHCR24-myc group exhibited much higher expression levels of Bip than control cells, especially at 48 h after TM exposure, suggesting that DHCR24 overexpression promoted the upregulation of Bip to enhance the protein folding capacity and prevent protein accumulation, thus alleviating ER-stress induced by TM.

Next, we further explored the role of DHCR24 on the UPR signaling pathway by investigating the activation of ATF6 and p38. As shown in Figures 3(a) and 3(c), ATF6 expression quickly increased in Ad-LacZ-infected cells and peaked three hours after TM exposure. In contrast, in the Ad-DHCR24-myc group, ATF6 expression gradually increased over the time of TM exposure (Figures 3(a) and 3(c)). Six hours after TM expression, levels of ATF6, including cleaved ATF6, were significantly higher in the Ad-DHCR24-myc group compared to the Ad-LacZ group. Conversely, the activation of p38 was significantly inhibited by infection with Ad-DHCR24-myc compared to the control adenovirus. These data indicated that DHCR24 overexpression may alleviate ER stress by enhancing the activation of ATF6 and inhibiting the activation of p38.

### 3.4. Overexpression of DHCR24 Scavenges Excessive Intracellular ROS Induced by TM in MIN6 Cells

**Figure 4:**
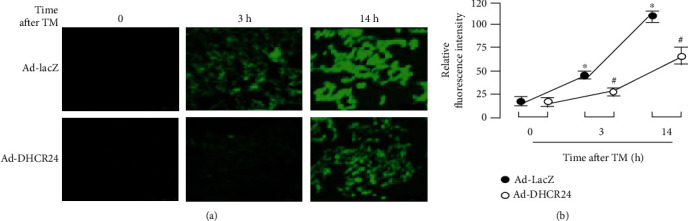
Excessive intracellular ROS is scavenged by overexpression of DHCR24. (a) Intracellular ROS were detected by application of the fluorescent dye DCFHDA. Bar, 50 *μ*m. (b) The levels of intracellular ROS are represented by the relative fluorescence of DCF (2′,7′-dichlorofluorescein). Mean ± S.D. (*n* = 3). ^∗^*p* < 0.05 vs. the levels at 0 h after TM treatment. ^#^*p* < 0.05 vs. Ad-LacZ.

There is a close relationship between ER stress and oxidative stress, and it has been shown that some antioxidants can reduce ER stress [31–33]. DHCR24, as an antioxidant, has been confirmed to have a protective function against ER and oxidative stress, attenuating neuronal apoptosis by clearing intracellular ROS under ER stress conditions [22]. Therefore, we performed a similar experiment in MIN6 cells to investigate whether adenovirus-driven overexpression of DHCR24 could scavenge ROS, which accumulate in pancreatic *β* cells during ER stress. As shown in Figures 4(a) and 4(b), by assessing intracellular ROS levels using the fluorescent probe DCFHDA, we observed that cells infected with Ad-LacZ exhibited significantly higher levels of fluorescence after TM treatment compared to baseline, suggesting that TM exposure induced the generation of intracellular ROS. Conversely, much weaker fluorescent signals were detected in the Ad-DHCR24-infected cells, indicating that DHCR24 overexpression was able to alleviate the oxidative environment induced by ER stress.

### 3.5. Upregulation of DHCR24 Protects MIN6 Cells against Oxidative Stress-Induced Apoptosis

**Figure 5:**
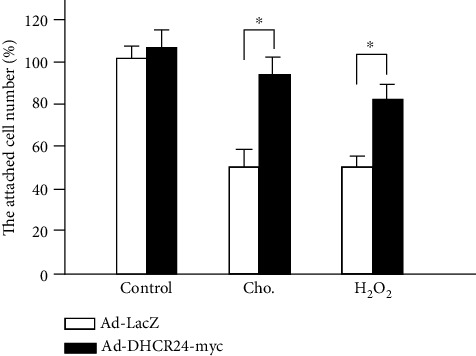
MIN6 apoptosis induced by both cholesterol and H_2_O_2_ is inhibited by overexpression of DHCR24. MIN6 cells were exposed to cholesterol (250× dilution) and H_2_O_2_ (1 mM) 48 h postadenovirus infection. The adherent cell number was calculated as percentage of cells infected with Ad-LacZ in the control group. Mean ± S.D. (*n* = 3); ^∗^*p* < 0.05 vs. Ad-LacZ.

Oxidative stress plays an important role in the apoptosis of pancreatic *β* cells. Previous studies have confirmed that cholesterol and H_2_O_2_ induce apoptosis in MIN6 cells by oxidative stress, which can be reversed by treatment with antioxidants [8, 34]. Therefore, to investigate whether DHCR24 could confer resistance to oxidative stress-induced apoptosis, we applied cholesterol and H_2_O_2_ to MIN6 cells as inducers of oxidative stress and quantified apoptosis. As shown in Figure 5, treatment with cholesterol and H_2_O_2_ induced an obvious loss of cells in the Ad-LacZ group. Infection with Ad-DHCR24-myc significantly increased the attached cell numbers exposed to cholesterol and H_2_O_2_, suggesting that upregulation of DHCR24 protected MIN6 cells against apoptosis caused by oxidative stress.

### 3.6. Downregulation of Endogenous DHCR24 Promotes Apoptosis of MIN6 Cells under ER Stress Conditions

**Figure 6:**
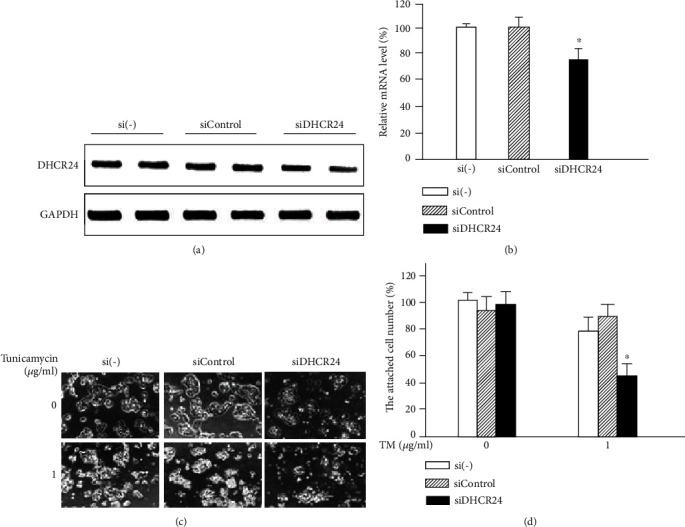
Downregulation of endogenous DHCR24 promotes MIN6 cell apoptosis caused by ER stress. (a) The mRNA levels of DHCR24 were measured by semiquantitative RT-PCR after infection of MIN6 cells with siControl and siDHCR24. (b) The relative mRNA levels of DHCR24 were normalized to GAPDH and calculated as percentage of the levels in the si(-) group. Mean ± S.D. (*n* = 3); ^∗^*p* < 0.05 vs. si(-) or siControl. (c) Images of MIN6 cells were taken with a phase-contrast microscope after infection with siControl and siDHCR24 followed by treatment with TM for 24 h. Scale bar, 100 *μ*m. (d) The adherent cell number was calculated as percentage of cells in the si(-) group at 0 h after TM treatment. Mean ± S.D. (*n* = 3); ^∗^*p* < 0.05 vs. si(-) or siControl.

Finally, we examined the impact of endogenous DHCR24 on MIN6 cells under ER stress conditions using siRNA interference. We confirmed the efficiency of siDHCR24 transduction using semiquantitative RT-PCR. Cells transfected with siDHCR24 showed a significant downregulation of the DHCR24 mRNA level (Figures 6(a) and 6(b)) compared to cells without siRNA transfection or cells transfected with siControl RNA, demonstrating that siDHCR24 successfully inhibited the expression of endogenous DHCR24 mRNA in MIN6 cells. Without TM treatment, transfection of cells with siControl or siDHCR24 had no obvious effect on the cell viability. However, 24 h after TM exposure, cells transfected with siDHCR24 were more sensitive to apoptosis, exhibiting a distinct loss of cells compared to cells without transfection and cells transfected with siControl (Figures 6(c) and 6(d)), suggesting that downregulation of endogenous DHCR24 reduced the capacity of MIN6 cells to resist apoptosis induced by ER stress. These results further demonstrated that DHCR24 could protect pancreatic *β* cells from ER stress-induced apoptosis.

## 4. Discussion

ER stress plays a key role in the apoptosis of pancreatic *β* cells in T2D, and protection of pancreatic *β* cells against apoptosis is an attractive strategy for the prevention and treatment of T2D. In the current research, we found that DHCR24 overexpression protected MIN6 cells against apoptosis caused by ER stress by enhancing Bip expression levels via potentiation of ATF6 activation and inhibition of p38 activation. This antiapoptotic role of DHCR24 in MIN6 cells may be closely related to its capability to scavenge excessive intracellular ROS in particular since DHCR24 overexpression could significantly inhibit TM-induced ROS accumulation and reduce MIN6 cell loss induced by oxidative stress. These findings indicate that DHCR24 could be considered a candidate target for T2D drug development.

There is accumulating evidence for a broad antiapoptotic function of DHCR24. DHCR24 was identified in the human brain by Greeve et al. and initially named as seladin-1 (selective Alzheimer's disease indicator 1) [20]. DHCR24 is significantly downregulated in neurons of vulnerable regions in the AD brain, and overexpression of DHCR24 protects neuronal cells *in vitro* (PC12 cells and H4 cells) against A*β* toxicity and oxidative stress. In a previous study, we demonstrated, for the first of time, that DHCR24 overexpression protects neuronal N2A cells against ER stress-caused apoptosis [22]. Taken together, these studies demonstrate a neuroprotective function of DHCR24. Furthermore, it has been confirmed that DHCR24 protects MEFs from apoptosis induced by H_2_O_2_ and ER stress through scavenging intracellular ROS [23]. Finally, the antiapoptotic activity of DHCR24 is also reflected by its abnormally high expression in tumor tissues [18, 35, 36], and studies have suggested that DHCR24 can be used as an indicator of poor clinicopathological characteristics of cancer patients [37]. Here, we describe, for the first time, an antiapoptotic role for DHCR24 in pancreatic *β* cells (Figures 1, 2 and 6), expanding the role of the antiapoptotic activities of DHCR24.

Pancreatic *β* cells are some of the body's most sensitive cells to ER stress due to the vital importance of the ER for insulin synthesis [5]. UPR is the earliest response to ER stress, characterized mainly by translational attenuation, upregulation of ER chaperone proteins, and proteasomal degradation of misfolded proteins. Prolonged and unresolved stress results in a transformation from adaptive to maladaptive or proapoptotic responses, which are usually related to pathological states [38]. Engin et al. have described that sXBP1 is markedly upregulated in the prediabetic stage of a high-fat diet- (HFD-) fed mouse model, indicating that the UPR response can be observed even after relatively mild dietary stress. Once HFD-fed mice progress to hyperglycemia, the expression of ATF6, an activating transcription factor contributing to the expression of ER chaperones to alleviate ER stress, is decreased significantly, hinting at an unsuccessful response of the *β* cell UPR to compensate for the sustaining insulin demands. Similarly, reduced expression levels of ATF6 and sXBP1 coincide with hyperglycemia in both a genetic mouse model (*ob/ob*) and humans with type 2 diabetes [38]. *β* cells are more susceptible to ER stress-induced apoptosis when ATF6 is knocked down and more resistant to apoptosis when overexpressing active ATF6*β* [39]. Interestingly, it has also been found that genetic variation in ATF6 is associated with prediabetes in the Chinese Han population [40]. These reports indicate that ATF6 may represent a therapeutic target for T2D.

In our study, MIN6 cells exposed to TM exhibited an upregulation of Bip protein levels, suggesting the presence of ER stress (Figure 3). ATF6 was activated strongly in cells infected with Ad-LacZ, peaking as early as 3 h after TM treatment, but then decreased sharply, indicating that these cells were unable to cope with the sustained TM stimulation. In contrast, cells infected with Ad-DHCR24-myc still showed significant and sustained upregulation of ATF6. Correspondingly, the expression of Bip, regulated by activated ATF6, was significantly higher in this group than in the Ad-LacZ group 48 h after TM exposure. In addition, DHCR24 overexpression also inhibited activation of the p38 cell signaling pathway (Figure 3). As a result, upregulation of DHCR24 rendered MIN6 cells more resistant to apoptosis caused by ER stress.

In response to incorrect disulfide bond formation and misfolded protein accumulation during ER stress, abundant ROS is produced, leading to an imbalance of the ER redox system [41]. In the present study, the production of ROS after TM exposure was much weaker in the Ad-DHCR24-myc group than in the control group (Figure 4), demonstrating that DHCR24 possesses the ability to balance the oxidative environment under ER stress conditions. Overexpression of DHCR24 has been shown to directly scavenge ROS generated during ER stress [23], therefore attenuating the impairment of ER stress and resulting in an upregulation of Bip protein levels and blocking the prolonged activation of p38. Taken together, our results suggested that overexpression of DHCR24 protected MIN6 cells from TM-induced apoptosis through scavenging excessive ROS.

Our data showed that DHCR24 overexpression significantly increased the attached MIN6 cell numbers exposed to cholesterol and H_2_O_2_ (Figure 5). Previous studies have confirmed that cholesterol and H_2_O_2_ induce apoptosis in MIN6 cells by generating excessive ROS, which can be reversed by reduced glutathione and the antioxidant cyanidin-3-glucoside [8, 34]. Combined with our data showing that DHCR24 inhibited the TM-induced ROS production, we hypothesize that the function of DHCR24 in protecting MIN6 cells from the cholesterol and H_2_O_2_-induced apoptosis may also stem from scavenging excessive ROS.

ROS is an intermediate connecting ER stress to oxidative stress, both of which are important pathological processes in insulin resistance [41, 42]. Mitochondria are the main source of ROS, and overproduction of ROS is related to an inflammatory response and mitochondrial dysfunction [41, 43]. Nuclear factor-kappa light chain enhancer of B cells (NF-*κ*B) and nuclear factor-erythroid 2-related factor-2 (Nrf2) are two redox-sensitive transcription factors that are coregulated in order to maintain the redox homeostasis in healthy cells. Activation of the NF-*κ*B signaling pathway is generally companied by a negative regulation of Nrf2 during the inflammatory responses in diabetes [44]. In skeletal muscle cells, inhibition of NF-*κ*B alleviates mitochondrial dysfunction, attenuates the enhanced ROS levels, and improves insulin sensitivity following sustained nutrient overloading [45]. It has also been found that a high-fat diet increases NF-*κ*B expression while decreasing Nrf2 levels in liver tissues of mice, which can be reversed by supplementation with docosahexaenoic and extra virgin olive oil [46–48]. Activated NF-*κ*B unidirectionally inhibits the transcriptional function of Nrf2 by either promoting the localization of transcriptional repressors or activating the cytosolic inhibitor Keap1 and subsequently leads to a decreased transcription of antioxidant defense enzymes and increases the accumulation of ROS [49–51]. McGrath et al. have demonstrated that upregulation of DHCR24 mediates the anti-inflammatory effects of high-density lipoprotein (HDL) in endothelial cells, while silencing of DHCR24 increases the expression of NF-*κ*B which could not be suppressed by reconstituted HDL [52]. Based on these evidences, we hypothesize that, in addition to directly scavenging ROS, overexpression of DHCR24 may attenuate increased ROS levels and improve the insulin sensitivity in insulin resistance models of T2D by downregulating the expression of NF-*κ*B and upregulating the expression of Nrf2. However, this hypothesis needs to be further investigated.

In addition to its role in ROS scavenging, the antiapoptotic activity of DHCR24 may also be associated with its role in cholesterol biosynthesis, since the cholesterol is required for the maintenance of caveolae/lipid raft structure and function in the plasma membrane, which is the localization of many growth factor receptors [53]. This is supported by one of our previous studies, showing that the infection of neuronal cells with Ad-DHCR24-myc elevates cholesterol levels and is accompanied by an increase in caveolin 1 and insulin-like growth factor receptor [22]. However, in the present study, the infection of MIN6 cells with Ad-DHCR24-myc had no significant effect on the intracellular cholesterol levels (Figure 1(b)), indicating that the antiapoptotic function of DHCR24 in MIN6 is predominantly accomplished by its scavenging of ROS. Moreover, these results suggest that the influence of DHCR24 on intracellular cholesterol level is cell type-specific, which might be due to differential regulation mechanisms of cholesterol homeostasis in different cell types.

## 5. Conclusion

In conclusion, although only a preliminary study, the current work provides clear evidence that DHCR24 protects pancreatic *β* cells from ER stress-induced apoptosis by scavenging excessive intracellular ROS. These results suggest that targeting DHCR24 may represent a new therapeutic strategy for T2D.

## Data Availability

The data used to support the findings of the present study are included within the article.
